# Stem Cell Treatment Trials for Regeneration of Testicular Tissue in Laboratory Animals

**DOI:** 10.1007/s43032-022-01152-1

**Published:** 2023-01-05

**Authors:** Hamdy Y. Ismail, Shaymaa Hussein, Nora A. Shaker, Hamdy Rizk, Y. R. Wally

**Affiliations:** 1 Department of Anatomy and Embryology, Faculty of Veterinary Medicine, Cairo University, 12211 Giza, Egypt; 2 Department of Cytology and Histology , Faculty of Veterinary Medicine, Cairo University, 12211 Giza, Egypt

**Keywords:** Infertility, Testicular degeneration, Stem cell, Chemotherapy, Laboratory animals

## Abstract

Infertility is a serious medical, economic, and psychological problem in the society. Male factor infertility, due to defective spermatogenesis as a result of a failure in germ cell proliferation and differentiation, appears to be the cause of 25–50% of infertility cases. According to several surveys, testicular degeneration can be caused by a variety of physical, chemical, and microbial causes. A stem cell is a non-specialized cell which is characterized by self-renewal by mitotic cell division and able to differentiate to specialized cells for the various tissues of the body. The data were obtained and analyzed from different databases (PubMed, Google Scholar, Egyptian Knowledge Bank, Elsevier, Medline, Embase, ProQuest, and BMC). This review discusses the causes, symptoms, and grades of testicular degeneration and the use of different types of stem cells in regeneration. And its conclusion based on previous researches and trials, MSCs are considered effective therapy for testicular degeneration.

## Introduction

About 60–80 million couples worldwide have problems of infertility and about 40–50% of these cases are caused by a male factor [1]. Testicular degeneration (TD) is defined as a process in which the structure of the testis deteriorates, resulting in the loss of testicular function, and TD can be caused by a variety of physical, chemical, and microbial causes [2].

At all stages of life, the testis has been demonstrated to be very vulnerable to the harmful effects of irradiation and chemotherapy. Male germ cells are especially sensitive to various kinds of chemotherapeutic agents, and the impact of combination chemotherapy on the spermatogenic epithelium depends on the type and quantity of the medications used [3]. The advances in cancer treatment give the chance for childhood and adult cancer patients to experience 5 years survival rate up to 82% [4]. Cancer treatment protocols focus on the treatment of the disease itself, but individuals place an emphasis on treatment safety and efficacy [5].

Chemotherapy and radiation therapy for cancerous patients are highly effective but their gonadotoxic side effects may have a negative effect on fertility; this depends on the chemical agent and the dose and may cause permanent or temporary gonadal damage in male patients [6]. Nearly 1% of all men have azoospermia which can be either obstructive or non-obstructive azoospermia and men that suffer from azoospermia represent 10 to 15% of all infertile cases [7].

There are many surgical and hormonal treatments in these cases but in the recent years, a great attention has been given to stem cell therapy [8]. From the moment the ovum is fertilized until death, these cells are present in every living organism. Their existence enables the body to grow and maintain a healthy amount of somatic cells. They also allow for the regeneration of organs and tissues by rebuilding deteriorated or damaged somatic cells [9].

This review’s objective is to investigate the effectiveness of mesenchymal stem cells from various sources in the treatment of male infertility. The findings of the present review may be crucial in determining how well MSC treatment is applied in clinical trials.

## Material and Methods

Data for our review was gathered from a variety of databases (PubMed, Google Scholar, Elsevier, Egyptian Knowledge Bank, Medline, Embase, BMC, ProQuest, etc.). The search focused on the laboratory animals: rat, mice, hamster, and Guinea pig at the last two decades on testicular degeneration, as well as therapy options including various types of stem cells and their clinical outcomes.

## Results

### Structure of the Rabbit Testes

Testis of the negative control group appeared as elongated ovals with a laterally compressed range from 2.8 to 3.2 cm in length with a sharp caudal pole and a blunt cranial pole located in the inguinal region inside two thin hairless scrotal sacs just cranial to the penis. The testes had a marbled appearance, slightly firm in consistency, with the tunica vasculosa running longitudinally on the ventral free border. The testes were characterized by centro-axial mediastinum toward the cranial pole [10].

Zamora E Jl and Felipe-Pérez Ye [11] mentioned that the tunica albuginea had the connective tissue septum that entered the testicular parenchyma, separating it into lobules partially or completely. Each lobule was made up of four to six seminiferous tubules, which was covered by stratified epithelium of spermatogenic cells and Sertoli cells. The spermatogonia are located in the basal membrane as tiny, oval, or spherical cells with various degrees of chromatin condensation in their nuclei. These cells formed primary spermatocytes, which undergo first meiotic division, giving secondary spermatocytes that had the second mitotic division swiftly producing spermatids. The authors added cells that produced testosterone found in the connective tissue between seminiferous tubules. These cells were known as Leydig cells and they had spherical nuclei and acidophilic cytoplasm.

Zamora E Jl and Felipe-Pérez Ye [11] recorded the number of germinal cells reduced at the end of the seminiferous tubules, but the number of Sertoli cells increased. The transition zone or segment that connected the seminiferous tubule to the straight tubule was lined entirely by Sertoli cells. Straight tubules appeared in a network of anatomical canals known as the rete testis, which can be lined by simple flat, cubic, or columnar epithelium.

### *Normal Parameters of Semen in Laboratory Animals (Table *1*)*

**Table 1:** Normal semen parameters in laboratory animals

	Rabbit	Rat	Mice	Guinea pigs
Count/concentration	210.5 × 10^6^/ ml in winter and 156.11 × 10^6^/ ml in summer	60.8 ± 0.20 × 10^6^ sperm/rat	106.5 ± 42.5 × 10^6^	60.00 ± 1.90 × 10^6^ /cauda epididymis 250.20 ± 5.40 × 10^6^ /g cauda epididymis
Motility	56% in winter and 40.6 in summer	48.4 ± 2.03%	Rapid motility grade A 20.18 ± 7.08% -Slow motility grade B 22.64 ± 5.0%	90.00 ± 5.35%
Viability/live sperm concentration	180.8 × 10^6^/ ml in winter and 129.9 × 10^6^/ ml in summer	85.1 ± 3%	75.09 ± 9.42%	**-**
Testosterone level	11.0 ± 0.02 ng/ml	2.76 ± 0.27 ng/ml	3.25 ± 1.582 ng/ml	1.2–1.4 ng/ml
Testis weight	3.60 ± 0.09 g	1.40 ± 0.821 g	659 ± 52 mg per 100 g of body weight	0.23 ± 0.01 g/100 g of BW)
Authors	[12, 13]	[14]	[15]	[16]

Castro, Berndtson [17] determined that plasma and testicular testosterone levels were correlated significantly with seminiferous tubular diameter, number of Sertoli cells per tubules cross sections, and ratio between germ cells and Sertoli cells. Both plasma and tissue levels of testosterone correlated highly with percentage volume of Leydig cell nuclei (0.82–0.78 respectively) and the number of Leydig cells per gram of testis (0.83–0.82 respectively). Plasma testosterone level correlated with the total number of Leydig cells per testis (0.71). High level was observed between % volume of Leydig cell nuclei and number of Leydig cell per gram (0.94) and less extent between % volume of Leydig cell nuclei and total number of Leydig cells per testis (0.60).

### Testicular Degeneration

TD is not a well-known disease that has a large economic impact. A significant percentage of morphologic abnormalities of spermatozoa, poor motility, a low number of normal sperm per ejaculate, and decreased testicular size are all symptoms of TD. TD can be unilateral or bilateral, depending on whether the underlying cause is confined such as a locally aggressive malignancy, or widespread [18].

### Causes of Testicular Degeneration

TD develops as a result of a recognized testicular trauma. Testicular injuries, heat, cold, radiation, toxins, or ischemia, certain dietary deficiencies, exogenous androgen injection, infection, autoimmune illness, sperm outflow blockages, and neoplasia are all possible causes of testicular injuries [2]. Several arthropod-borne viruses can also cause testicular degeneration [19]; several cases of TD and testicular atrophy have been reported as a result of heavy metal exposure [20]. Microbial orchitis can range from a little infection of the affected testis to significant suppuration or organ necrosis, and can be caused by an ascending infection, hematogenous microbial dissemination, or direct microbial penetration into the organ. It frequently occurs in conjunction with epididymitis or as a result of pre-existing traumatic, viral, or parasite injury [21]. One of the major causes of testicular toxicity and degeneration at all stages of life is chemotherapy and radiation therapy [3], testicular degeneration can be classffied acording to the number of affected seminiferous tubules [22] (Table 2).

**Table 2:** Grading of testicular degeneration conducted on male albino Wister rats [22]

Grade	Number of affected tubules
Grade 1	< 10%
Grade 2	10–25%
Grade 3	25–50%
Grade 4	50–75%
Grade 5	> 75%

#### Chemotherapy

Chemotherapy and radiation therapy both cause germ cell death, resulting in oligo- to azoospermia and testicular atrophy. The type of therapy (especially alkylating drugs), treatment length, intensity, and drug combination are all important factors in determining the extent and duration of testicular harm. Chemotherapy-induced testicular damage appears to differ depending on the patient’s age at the time of treatment [23].

Chemicals are used to treat a wide variety of cancers. However, these drugs carry the potential of causing ovarian or testicular damage. Testicular cells are particularly sensitive since they go through a number of processes (e.g., mitotic, meiotic, synthetic, morphogenic) that chemotherapeutic agent’s target [24]. Although gonadal dysfunction may be transitory after therapy, recovery is generally unpredictable, and damage is permanent in some patients [25].

Chemotherapeutic drugs have enhanced great effect in cancer treatment. All anti-cancer drugs may cause permanent or temporary damage on different organs [26–28].

Chemotherapeutic drugs are classified into five main groups: alkylating agents such as cisplatin, cyclophosphamide, procarbazine, anti-metabolites such as methotrexate and 5-fluorouracil, antibiotics such as adriamycin, bleomycin, and mitoxantrone, antimicrobials such as vinblastine and vincristine, enzymes such as L-asparaginase [29].

Tumors are characterized by uncontrolled cell division so the chemotherapeutic drugs affect tumors by stopping cell division but this mechanism cannot differentiate between cancerous and normal cells causing damage and destruction to normal cells and tissues by apoptosis after chemotherapy [30–34].

Cisplatin is considered an important platinum-based chemotherapeutic drug which is used in the treatment of many tumors [35] but despite its high efficacy, it has major side effects in many organs such as the kidney and testis [36–38] In many animals models, cisplatin caused reproductive imbalance including germ cell depletion and testicular atrophy [36–39], also Leydig cell dysfunction and testicular steroidogenic disorder [40].

Animals treated with cisplatin suffer from decrease in fertility represented by decrease total number of sperms and decrease in semen viability and motility also increase in percentage of abnormal sperms [3].

##### Pathophysiology of Testicular Degeneration

The chemotherapeutic drugs such as alkylating agents especially cisplatin can cause oxidant/antioxidant imbalance, which lead to oxidative damage of many cellular elements like DNA, proteins, and lipids [36, 37, 41]. Antioxidant defense systems such as catalase (CAT), superoxidedismutase (SOD), and reduced glutathione (GSH) are considered the protecting mechanism in tissues against the reactive oxygen species (ROS) damage [42]. Reactive oxygen species are routinely produced in the mitochondria of the testes and subsequently scavenged by the antioxidant defense systems [43]. However, several substances, such as cisplatin, can disrupt this balance by disrupting the pro-oxidant–antioxidant balance, resulting in cell malfunction [44].

The exposure to cisplatin leads to excessive production of free radicals, which alters the bio membranes and causes severe damage [45]. Low levels of ROS are suggested to have a positive effect on fertility by enhancing sperm maturation processes like capacitation but in cases of cisplatin exposure, its levels increase that causing impairment of fertility and embryo development [46].

In rat, [47] mentioned that testes injected with cisplatin had considerable reduction in weight and size of testis and decrease in the daily sperm production as well as the percentage of viable and motile sperms. Moreover, the testosterone levels decreased in cisplatin-treated groups in comparison with normal rats (Sherif et al. 2018).

Meligy, Abo Elgheed [1] showed that the H&E sections of testes in rat taken from cisplatin-treated groups had severe distortion of most seminiferous tubules which observed the reduction on germinal epithelium thickness with irregular empty spaces and widely separated, with few or no sperms seen. The germinal epithelium was deficient in other tubules with development of multinucleated giant cells. Vacuolation and exfoliation of germ cells were also seen, congested blood vessels were found in the interstitium, with a sub capsular congested blood vessel, and the testicular capsule becomes thicker and more irregular.

In rabbit, the administration of cisplatin resulted in a marked elevation in the levels of malondialdehyde (MDA) and reduction of testis antioxidant enzymes represented by GSH and glutathione peroxidase, CAT activity compared with control animals (Benzer et al. 2011).

Sherif, Sabry [48] recorded that the effect of cisplatin exposure on the cellular stress markers in rat testes showed a significant increase in testicular malondialdehyde by 142.7% and decrease in both reduced glutathione by 42.1% and superoxide dismutase by 56.9% levels compared to normal rats.

Benzer et al. (2011) observed that the MDA, which formed as a final product of the peroxidation of lipids, served as an index of the intensity of oxidative stress. The testes’ MDA levels were increased in cisplatin-treated group (about 10–12 nmol/g) when compared to the control one (4–6 nmol/g).

Faria et al. (2007) mentioned that certain enzymes catalytically eliminate free radicals and other reactive substances; this provides endogenous protection against oxidative stress. Catalase and glutathione peroxidase were among these enzymes.

Reddy, Madhu [47] reported that cisplatin treatment in the testes of rats proved a major decrease in activities of superoxide dismutase and catalase with an increase in the levels of H_2_O_2_ and lipid peroxides.

Benzer et al. (2011) recorded that the activity of catalase enzyme was decreased on cisplatin administration group (60–80 k/g protein) compared to control animals (120–140 k/g protein), where *k* is the first-order rate constant. Also, they added that glutathione peroxidase activity was reduced on group treated with cisplatin as (15–20 IU/g protein) from the normal level (30–35 IU/g protein). Moreover, they noted the glutathione was considered a compound involved as coenzyme in oxidation–reduction reaction, which its level was decreased in cisplatin-treated group (1.75–1.80 nmol/g) that differs from control group (1.80–1.90 nmol/g).

### Stem Cell Therapy

Stem cells have extensive renewal ability and produce daughter cells that undergo further differentiation [49]. These cells have many sources such as bone marrow, peripheral blood, dental pulp, hair follicle, and adipose tissue which is considered one of the easiest sources of stem cells isolation [50–52]. Also, Wharton’s jelly, which is isolated from umbilical cord blood and tissue, is used to isolate the most primitive mesenchymal stem cells (MSCs) (Tables 3, 4, 5, 6, and 7) [9, 53].

**Table 3:** Summary of rats’ bone marrow mesenchymal stem cells (BM-MSCs) research

Author Year Reference	Model	Source and dose	Route and timing	Main findings
Elbaghdady, Alwaili [68]	Sprague–Dawley rats (cadmium-induced testes’)	BM-MSCs First dose (1 × 10^6^) At induction Second dose (1 × 10^6^) after 1 week	I/V	- When compared to the cadmium-treated rats (Cd group), the stem cell–treated group gained more weight - When compared to the Cd group, the treated group had considerably higher sperm count and viability - The histopathology of the (Cd group) indicated total testicular atrophy. These characteristics were not seen in animals treated with stem cells
Mohamed, Mohamed [69]	Adult male Sprague–Dawley rats (gentamycin induced testis)	BM-MSCs dose 10 μl of cell suspension	Intra-testicular 2 weeks after gentamycin induction	- Compared to control rats, gentamicin injection for 2 weeks resulted in a significant drop in serum testosterone, intra-testicular testosterone, and estradiol at the end of the second, sixth, and tenth weeks - At the end of the second, sixth, and tenth weeks, gentamicin injection for 2 weeks resulted in a significant increase in the percentage of fragmented DNA in testicular tissue as compared to control rats - At the conclusion of the 6th and 10th weeks, stem cell therapy increased blood testosterone and testicular testosterone and estradiol levels in testicular tissue, protecting the testis from gentamicin toxicity. Following a gentamicin injection for 2 weeks -At the conclusion of the 6th and 10th weeks following gentamicin injection for 2 weeks, stem cell therapy resulted in a considerable reduction in the percent of fragmented DNA in testicular tissue and considerable rise in sperm count and viability
Sherif, Sabry [48]	Male Sprague–Dawley rats (Cisplatin-induced gonadotoxic)	BM-MSCs dose (2 × 10^6^)	I/V On the next day after induction	- When compared to control rats, the cisplatin group showed considerable weight loss in the testis. The infusion of BM-MSCs resulted in a considerable increase in testicular weight - In comparison to normal rats, rats injected with cisplatin alone showed a substantial increase in testicular MDA and depletion in both GSH and SOD levels. Rats injected with BM-MSCs, on the other hand, showed a considerable reduction in oxidative stress levels, as evidenced by a significant decrease in MDA testicular levels and a significant increase in GSH
Zhang, Liu [70]	Male Sprague–Dawley rats Busulfan-induced azoospermic rat	BM-MSCs Dose 10^5^ cells	Injected into the seminiferous tubules of the recipient rats 4 weeks after Busulfan injection	- In vivo, rat BM-MSCs were transplanted into the seminiferous tubules of Busulfan-treated infertile rats after being co-cultured with Sertoli cells in a trans-well method and in conditioned media in vitro. This offered a suitable spermatogenic milieu (niche) in which they could be tested to see if they could trans differentiate into sperm cells - BM-MSCs are appropriate for allogeneic transplantation since they are hypo-immunogenic and induce no immunosuppression or immune-surveillance after transplantation. Sertoli cells are also immune-tolerance cells. This helps the donor BM-MSCs survive in the recipient seminiferous tubules,
Monsefi, Fereydouni [66]	Wistar male rats sterilized with Busulfan injection	RAT (BM-MSCs) Dose 1.75 × 10^5^	Injected into testis	- After 2 months, histological studies of testes in the Busulfan treatment group indicated degenerative alterations in the majority of tubules, including seminiferous tubular atrophy and germinal epithelium degradation. Some tubules had fewer sperm clusters and spermatids, while spermatogonia and primary spermatocytes were of normal size but had a much lower number - In their new environment, MSCs transformed into testicular germinal cells. MSCs also developed into Leydig-like cells seen between testicular seminiferous tubules - Morphometrical testis revealed that the width of the seminiferous tubules, as well as the number of cells in the MSC injected group, was not significantly different from the control group
Hassan and Alam[42]	Adult male albino rats (Lead nitrate–induced infertility)	Rat BM-MSCs Dose 1 × 10^6^ MSCs	1 week After a single dose of LN	- In comparison to the control group, LN caused a statistically significant rise in testicular MDA content and a statistically significant decrease in SOD, CAT, and GPx activity at various time intervals after exposure - When compared to the lead-only group, treatment with MSCs after LN resulted in a considerable decrease in MDA levels and a significant increase in SOD, CAT, and GPx activity - LN caused a significant decline in testosterone level - MSCs prevented the drop in serum testosterone levels after 21, 30, and 60 days, bringing them closer to control levels - The mean percentage of sperm count and sperm motility in LN-treated mice was significantly lower than in control animals - After being exposed to LN, there was a considerable rise in sperm shape anomalies - MSC treatment enhanced sperm count and motility considerably

**Table 4:** Summary of rats’ ADMSCs research

Author Year Reference	Model	Source and dose	Route and timing	Main findings
Cakici, Buyrukcu[67]	Male Wistar rats (Busulfan-induced testis)	ADMSCs	Intra-testicular After induction of infertility	- The spermatogenesis process was stopped after treatment with Busulfan - Following the Busulfan treatment, samples without ADMSCs, scanning of sections revealed atrophy, complete and incomplete spermatocytic arrest, and a Sertoli cell–only appearance - In portions of stem cell–treated tissues, however, the tubules looked to be filled with spermatogenetic cells, but at a low rate. The presence of spermatozoa was discovered
Atalla, Saleh [71]	Male albino rats (Calcium chloride–induced testis)	ADMSCs Dose (10^6^ cells)	Intra-testicular After 1 week from cacl2 injection	- The remaining indigenous spermatogenic stem cells in the testis respond to ADMSCs as a stimulatory agent - OCT4, SOX2, Rex1, and FoxD3 are pluripotent markers that affect spermatogenesis were expressed by adipose tissue–derived mesenchymal stem cells (ADMSCs) - ADMSCs may secrete significant quantities of vascular endothelial cell growth factor, which inhibits apoptosis, insulin-like growth factor-1, which promotes stem cell proliferation and hepatocyte growth factor, which inhibits apoptosis These released cytokines may stimulate the expression of mRNA and proteins in the testes, allowing them to heal
Meligy, Abo Elgheed [1]	Adult male albino rats (Cisplatin-induced testicular damage)	ADMSCs Dose (1 × 10^6^)	Intra-testicular 5 days after induction	- Cisplatin-treated testis showed a significant change in structure, with the seminiferous tubules becoming twisted and the germinal epithelium’s thickness decreasing up to being depleted. Germinal epithelium depletion, with the exception of germ cells in the basal compartment, was attributed to germ cell sloughing and basal compartment capacity decrease - MSCs successfully restored testicular tissue and function - The testicular structure of the stem cell–treated group was significantly improved when compared to Cisplatin-treated groups, according to light and electron microscopic results from this study. In addition, the hormonal tests revealed that testosterone levels had returned to normal

**Table 5:** Other types of stem cells in rats

Author Year Reference	Model	Source and dose	Route and timing	Main findings
Hussein, Mohamed [41]	Male Wistar rats (Cisplatin-induced testicular toxicity)	Spermatogonial stem cell (SSC) Dose 1 × 10^6^	Intra-testicular On the 6th day after CP injection	- CP treatment alone resulted in a considerable rise in MDA levels as compared to the control group. The injection of stem cells to CP-treated rats decreased the elevated MDA levels - In CP-treated rats, there was a considerable decrease in CAT and GSH-Px activity compared to controls, which was significantly restored by stem cell treatment - The testicular weight, diameter of seminiferous tubules, height of the germinal epithelial lining of the seminiferous tubules, sperm count, and motility of male rats treated with CP all decreased significantly when compared to control whereas Concurrent treatment of stem cells with CP improved all parameters and reduced CP's harmful effects
Hsiao, Ji[72]	Sprague–Dawley rats (testicular torsion–induced germ cell injury)	MSCs from human orbital Fat tissues (OFSCs) Dose 3 × 104	Intra-testicular 2.5 h after torsion	MSCs provide therapeutic advantage by preventing testicular apoptosis, lowering intra-testicular oxidative stress, and increasing testosterone secretion, which protects spermatogenesis from torsion-induced germ cell injury - The majority of donated cells surround Leydig cells and release stem cell factor to aid spermatogenesis, although others may develop into Leydig cells

**Table 6:** Summary of mice stem cell research

Author Year Reference	Model	Source and dose	Route and timing	Main findings
Fang, Chao[73]	Wild-type Kunbai mice (Buslfan-induced testis)	MSCs were isolated from the bone marrow of male dairy Goat fetuses at 3^rd^ month gestation Dose 20 μl of the gMSCs (30 to 50 × 10^6^ cells /ml	Via the efferent ductules of the testis	- Goat MSCs exhibited male germ cell and spermatocyte markers, indicating that they had the ability to develop into male germ cells and aid spermatogenesis in endogenous germ cell-depleted patients via xenotransplantation - This shows that these cells provide a new source of male germ cells that could be exploited in the creation of male germ cells for various reproductive investigations
Abd Allah, Pasha[74]	Mice (Buslfan-induced testis)	Human umbilical cord blood stem cells and UCB-MSCs Dose 1 × 10^5^ cells	Intra-testicular	- After mice were given Busulfan via interperitoneal injection to induce azoospermia, their testes showed severe shrinkage, deformed seminiferous tubules (most of which did not contain sperms), and widely separated spermatogenic cells, indicating degeneration - The normal architecture of the testis was restored after MSC transplantation, according to histological inspection of the tissues. A thin connective tissue capsule encased it. The stratified germinal epithelium lined the seminiferous tubules, which were tightly packed - In the group treated with HSCs, despite enhanced vascularity indicated by congested blood arteries, most seminiferous tubules still revealed dilated lumens not containing sperms with a substantial drop in spermatogenic cells

**Table 7:** Hamster and Guinea pig stem cell research

Author Year Reference	Model	Source and dose	Route and timing	Main findings
Vahdati, Fathi [75]	Hamsters (Buslfan-induced azospermia)	BM-MSCs Dose (10^6^ cells)	Injected into the lumen of the seminiferous tubules 35 days after the last Busulfan injection	- Spermatogenesis could be induced by injecting BM-MSCs - Transplantation of BMSCs into hamster seminiferous tubules resulted in fast healing of pathological abnormalities in testicular tubules - Because BM-MSCs are hypo-immunogenic and have immunosurveillance or immunosuppressive qualities, they may be a good candidate for allogeneic cell transplantation
Hajihoseini, Vahdati [76]	Male outbred Dunkin–Hartley guinea pigs induced azospermia by Busulfan	BM-MSCs Dose (10^6^ cells)	Injected into the lumen of the seminiferous tubules 35 days after the last Busulfan injection	- BM-MSCs injection in Busulfan-induced azoospermic guinea pigs could induce spermatogenesis - Busulfan-treated Guinea pigs were examined histo-pathologically before and after receiving bone marrow–derived mesenchymal stem cells (BM-MSCs). The absence of germinal layer cells in the seminiferous tubules of Busulfan-treated testes indicates the spermatogensis; however, most seminiferous tubules appeared to have spermatogenic cells after treatment with BM-MSCs

The main purpose of any stem cell therapy is repair damaged tissues that do not have the ability to heel by itself; this gives hope to many patients to cure their diseases and replace dying cells [54].

MSCs’ therapeutic function is based on their ability to differentiate into cells that need to be replaced, as well as their immunomodulatory and paracrine activities, also their antioxidant effect  which aid in the healing of diverse tissue [53, 55].

Stem cells can be classified by the extent of its differentiation into four main cell types. These are totipotent, pluripotent, multipotent and unipotent. **Totipotent** have the ability to differentiate into all cell types as zygote cell which is formed at egg fertilization. **Pluripotent cells**, such as embryonic stem cells and cells derived from the mesoderm and endoderm, have the ability to differentiate into nearly all cell types. **Multipotent** have ability to differentiate into a related family of cells as adult stem cells that can become red and white blood cells or platelets. **Unipotent** have the ability to only produce cells of their own type as (adult) muscle stem cells [9, 53, 54].

Prządka, Buczak [9], Vikartovska, Humenik [53], and Kalra and Tomar [54] added other classifications according to the source of stem cells into two types: **early or embryonic stem cells** (ESCs) which is found in the inner blastocyst cell mass and **adult stem cells** (ASCs) which is found in adult body tissues.

MSCs are characterized as allogeneic, autologous, or xenogeneic cells depending on the donor–recipient relationship. Allogeneic cells come from a donor and are transplanted into a recipient of the same species. Autologous cell transplant is conducted on the same person and necessitates postponement of the process, whereas xenogeneic cells are transplanted from a donor who is not of the same species as the recipient [9].

One of the main features of stem cells is its ability to give rise to specialized cells by a process called differentiation which is controlled by signals inside and outside the cells; the internal signals are controlled by genes inside the cell while the external signals include chemicals which are secreted by other cells. In the recent years, stem cell biology has been focused on the antioxidant effect and its application in the repair of tissue damage caused by ROS [42, 56].

Roushandeh, Bahadori [57] mentioned that MSCs are type of multipotent stem cells isolated from tissues as bone marrow, fat, and amniotic membrane. [42, 53, 58, 59] observed that mesenchymal stem cells are characterized by specific surface antigen expression and their osteogenic, chondrogenic, and adipogenic differentiation ability and had the facility to self-renew.

The therapeutic ability of MSCs is based on its anti-inflammatory, anti-fibrotic, regenerative abilities; all of this could improve damages and degeneration in tissues [60]. Moreover, Fazeli, Abedindo [61] added that it was used in the treatment of many diseases such as healing of wounds, neurological and lung diseases, diabetes, cystic fibrosis, asthma, and cases of infertility. Lodi, Iannitti [62] noted that even if the use of MSCs has great therapeutic abilities, their uses in therapy are associated with some concerns as stem cells have the same renewal ability and plasticity with cancer cells; this may cause tumor development.

Adipose-derived stem cells (ADMSCs) are considered an excellent source of multipotent adult stem cells as their retrieval is very easy, also their isolation from subcutaneous adipose connective tissue and lipoaspirate [9, 63]. In addition, they have great proliferative ability producing huge number of cells and can be expanded for longer period of time [9, 64].

MSCs have the ability to protect the testis by different mechanisms; [8] cited that they have antioxidant and ROS-scavenging properties. [65] noted that MSCs have the ability to modulate the immune and inflammatory status caused by the cisplatin administration and he added that they have anti-apoptotic effects due to the over expression of the key anti-apoptotic protein Bcl2. Moreover, [66] mentioned that MSCs promote tissue regeneration by releasing growth factors and cytokines that encourage remaining spermatogonic cells to proliferate and finish their division.

Meligy, Abo Elgheed [1] reported that the histological sections and electron microscope results showed great enhancement in the testicular structure of groups treated with (1 × 10^6^) suspension of adipose-derived stem cells after cisplatin treatment compared to untreated groups. Also, groups which have been treated with stem cells showed normal levels of testosterone when compared to untreated groups.

The fertility that will be returned in stem cells treated groups may be either for the effect of stem cells in maintaining remaining spermatogonia stem cells in testis or that stem cell has the ability to differentiate into spermatogonia stem cell like cells which later form sperms [67].

Preclinical studies using mesenchymal stem cells for the treatment of testicular degeneration

## Conclusion

Infertility is a serious medical, economic, and psychological problem in the society; one of the main causes of infertility among males is testicular degeneration with chemotherapeutic agents. The animal model was most widely used for stem cell treatment trials with a satisfactory therapeutic result; MSCs have proven to have extensive renewal ability based on its ability of differentiating into cells that needs to be replaced. Finally, we can say based on previous researches and trials, MSCs area considered effective therapy for testicular degeneration. The development of stem research together with using 3D culture, exosomes, and scaffold delivery systems may increase the hope in getting most benefits from stem cell treatment of testicular degeneration.

## Data Availability

This published review includes all data gathered or analyzed during the study.

## Abbreviations

ADMSCs: Adipose-derived mesenchymal stem cells
ASCs: Adult stem cells
BM-MSCs: Bone marrow mesenchymal stem cells
CAT: Catalase
CP: Cisplatin
ESCS: Embryonic stem cells
GSH: Reduced glutathione
GSH-Px: Glutathione peroxidase
MDA: Malondialdehyde
MSCs: Mesenchymal stem cells
OFSCs: Orbital fat stem cells
ROS: Reactive oxygen species
SC: Stem Cells
SOD: Superoxide dismutase
SSCs: Spermatogonial stem cell
TD: Testicular degeneration
UCB-MSCs: Umbilical cord blood-mesenchymal stem cells
